# The effects of swing assistance in a microprocessor-controlled transfemoral prosthesis on walking at varying speeds and grades

**DOI:** 10.1017/wtc.2023.4

**Published:** 2023-03-02

**Authors:** Jantzen Lee, Michael Goldfarb

**Affiliations:** Mechanical Engineering, Vanderbilt University, Nashville, TN, USA

**Keywords:** control, human–robot interaction, mechatronics, prosthetics

## Abstract

This article proposes, describes, and tests a swing-assist walking controller for a stance-controlled, swing-assisted knee prosthesis that aims to combine benefits of passive swing mechanics (e.g., quiet operation, biomimetic function, and low power requirements) with benefits of powered swing assistance (e.g., increased robustness of swing-phase motion and specifically increased toe clearance). A three-participant, multislope, multispeed treadmill walking study was performed using the swing-assist prosthesis and controller, as well as using the participants’ prescribed microprocessor knee devices. The swing-assist device and approach were found to improve user minimum foot clearance during walking at slopes and speeds, and also to improve symmetry of knee motion. Hip power inputs from stance knee release to heel strike indicated that, on average, less hip power was required when using the swing-assist prosthesis, indicating that the observed benefits were likely the result of the knee device and its control methodology, rather than a result of increased hip joint effort.

## 1. Introduction

Trips and falls are a common cause of injury (Tinetti et al., 1994; Chang et al., 2016). For persons with lower limb amputation, the risk of falling is an even more prevalent issue (Miller et al., 2001), both in terms of fall frequency and incidence of negative health impacts (Kulkarni et al., 1996; Gauthier-Gagnon et al., 1999; Mundell et al., 2017; Kim et al., 2022). A major factor that can lead to falls during walking is incomplete knee extension at the end of swing phase, which reduces the stability of the knee during stance knee loading (Eveld et al., 2022), and often results in a less stable subsequent step, or potentially in knee buckling and a subsequent fall. Such incomplete knee extension can occur in non-perturbed walking, although it is certainly exacerbated by swing-phase perturbations such as scuffing the ground, wherein the bottom of the foot or shoe contacts the ground prior to the end of swing.

When walking with an energetically passive prosthesis, swing-phase knee motion results indirectly from active movement of the thigh segment, which generates knee movement via inertial coupling between the thigh and shank (Mochon and McMahon, 1980). Knee motion resulting from this ballistic swing is well suited to many walking activities, particularly level-ground and shallow-slope walking at moderate or fast speeds. At slower speeds, however, or on moderate or higher-grade slopes, net-zero or net-negative power at the knee joint cannot produce adequate knee motion, and therefore passive prostheses are limited in their ability to restore sufficient knee trajectories (and corresponding toe clearance) in these activities. The resulting insufficient knee movement increases the proclivity for scuffing (Begg et al., 2006; Rosenblatt et al., 2017) and incomplete knee extension prior to heel strike. Note also that the likelihood of scuffing the ground is made worse in prosthesis users due to the lack of ankle dorsiflexion in conventional prosthetic feet (Rosenblatt et al., 2014; Bartlett et al., 2021). This is especially problematic on inclines and non-even terrain, where healthy individuals more ably adapt to increase toe clearance (Gates et al., 2011).

The deficiency in swing-phase knee motion, especially at slower speeds and steep upslopes, can potentially be addressed with the addition of power at the knee joint. Specifically, knee movement can be supplemented with active power, particularly during slow and upslope walking, to increase toe clearance during these activities, and to help ensure full knee extension at the termination of swing phase. Several researchers have developed powered knee prostheses, which employ some type of swing-phase controller to provide swing-phase motion (e.g., Lawson et al., 2014; Azocar et al., 2018; Lenzi et al., 2018; Elery et al., 2020; Ghillebert et al., 2021). Although such approaches increase the robustness of swing phase, powered devices generally entail a higher output impedance relative to passive devices (due to reflected friction and inertia across the transmissions), and this higher output impedance generally sacrifices the ability to provide the (unpowered) ballistic swing phase that is characteristic of level-ground and shallow-slope walking.

In this article, the authors assess the prospective effects of a swing-assist knee prosthesis, as previously described in Lee et al. (2020), on the swing phase of walking at varying speeds and slopes. The swing-assist prosthesis utilizes a hydraulic-fluid-based cylinder, as employed in several microprocessor-controlled knee prostheses (MPKs), which modulates passive resistance via a controllable valve, in combination with a small swing-assist motor. Since the prosthesis employs a small motor utilizing a small transmission ratio (relative to a typical powered prosthesis), the prosthesis can provide sufficient knee torque and power to assist swing-phase motion, without significantly increasing the reflected impedance (i.e., inertia or friction) relative to a standard MPK, and therefore can allow ballistic swing phase when appropriate. A swing-assist approach has also recently been described by other researchers (Park et al., 2016; Baimyshev et al., 2018; Gao et al., 2019; Bartlett et al., 2022) as discussed subsequently.

To assess the prospective value of a swing-assist approach, this article describes a swing-assist controller intended to enhance the robustness of swing phase in walking and describes an experimental assessment of the effect of this swing-assist approach on the swing-phase characteristics of three transfemoral prosthesis users across varying walking speeds and slopes, relative to each user’s respective prescribed prostheses.

## 2. Swing-assist prosthesis prototype

**Figure 1.**
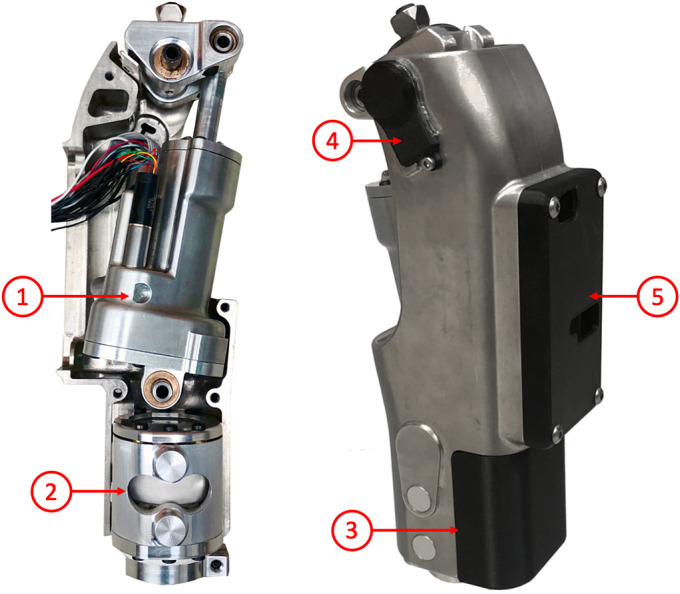
The assembled stance-controlled, swing-assisted knee prototype. The device consists of the actuator (1), load cell (2), battery pack (3), absolute encoder (4), and embedded system (5).

The Vanderbilt stance-controlled swing-assist (SCSA) knee, shown in Figure 1, is a transfemoral prosthesis prototype that consists of two parallel actuation systems: a high-power (dissipative) hydraulic system and a low-power (four-quadrant) electromechanical drive system. The hydraulic system consists of a two-way servovalve that provides resistance to movement, in parallel with a check valve. The check valve ensures that hydraulic resistance against extension is always low, even in the presence of high resistance against flexion. The active drive system consists of a motor that is coupled to the hydraulic piston via a lead screw. This system allows power to be bidirectionally added to the knee, and enables fine-tuning of bidirectional damping via passive motor braking. The prosthesis utilizes a suite of sensors, including an inertial measurement unit (IMU), load cell, absolute encoder at the knee joint, and an incremental encoder within the actuator. A detailed description of the prototype design is given by Lee et al. (2020).

## 3. Swing-assist controller

A recent review of knee-prosthesis control strategies describes a number of different control approaches to providing knee-prosthesis control (Fluit et al., 2020). Passive prostheses employ a ballistic swing phase, and generally modulate resistance to motion to prevent excessive knee flexion during fast walking. Powered prostheses, due to their higher reflected output impedances, generally employ some type of forcing function to drive swing-phase motion. Of note, the combination of ballistic swing capability with powered assistance is a new and emerging subgroup of prosthetic devices, and as such best practices do not yet exist for the control of this type of device. Existing swing-assist control methods for supplementing swing phase include position trajectory control with an emulated damping (Baimyshev et al., 2018); PD trajectory control (Park et al., 2016); constant feedforward torque as a flexion and/or extension aid (Gao et al., 2019); and a proportional control approach where the proportional gain varies as a function of a thigh-angle-based phase variable (Bartlett et al., 2022).

This article describes an alternative approach to swing-assist control, relative to the aforementioned ones, which is different in several essential ways. First, the sign (i.e., direction) of torque assistance is not a function of trajectory tracking error, but rather a function of stride time. This guarantees a single torque zero crossing, which promotes smooth assistance, and also facilitates a desired phasing of flexion relative to extension in swing phase. Second, swing assistance is only employed if the amplitude of motion is less than a minimum reference trajectory. When the motion exceeds the minimum reference (i.e., when it is more flexed during the flexion portion of swing, or when it is more extended during the extension portion of swing), no assistance is employed. Note that it is assumed that if excessive swing-phase flexion (i.e., the case when knee motion exceeds the reference by a large margin) were to occur, the excessive flexion would be corrected by the resistive control system (i.e., by a nominal setting of the hydraulic damping), as is the case with typical MPK prostheses.

**Figure 2.**
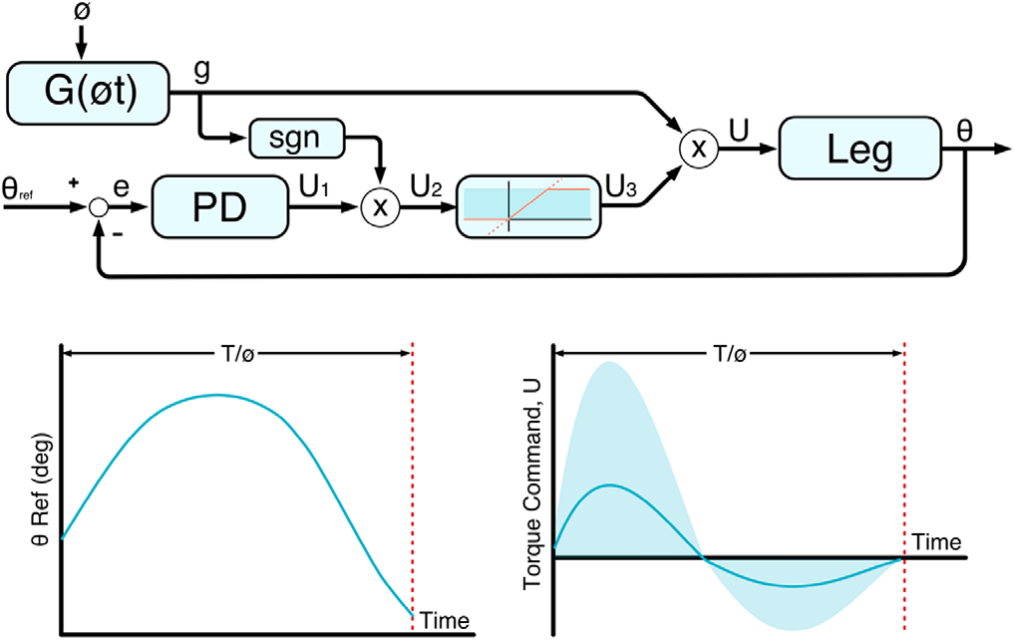
Swing-assist controller block diagram. Diagrammatic representation of the swing-assist control approach, where the reference angle and *ø* are generated within the external finite-state machine (FSM). An example plot of minimal acceptable trajectory ( 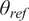 ) is shown in the bottom left, where the start angle and slope are a function of the user’s knee movement at toe off, and the peak angle is determined by cadence. This trajectory is compared against sensor signals to generate an error term (*e*), which is fed into a controller to generate a feedback term (*U*_1_). This term is then sign modulated based on the feed-forward term (*g*) to produce a sign-specific feedback (*U*_2_), which is saturated (*U*_3_), and applied as a gain to the feed-forward term to produce a torque command (*U*), which is sent to the leg. An example plot of the torque command is shown on the bottom right, where the solid line indicates *G*(*øt*) with a gain of 1, and the semitransparent region shows the possible envelope of real-time torque command values of *U.*

The structure of the swing-assist controller is shown in the block diagram of Figure 2. The controller employs a feedforward torque pulse, *g*, which is the output of a function of *G*(*øt*), constructed as a piecewise function of two fourth-order polynomials, as shown in the bottom right of Figure 2. In this function, *ø* is a temporal scaling factor generated at each toe off based on cadence, which shortens or lengthens the duration of swing phase, and *t* is the time since toe off. Additionally, upon toe off, a knee trajectory is generated using a spline function which is based on cadence, knee angle at toe off, knee angle velocity at toe off, and desired peak knee angle. This trajectory, shown diagrammatically as 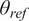 is used to generate a knee angle error, which is the input to a proportional derivative controller. Unlike a conventional trajectory controller, however, this trajectory represents a minimum acceptable trajectory; that is, if the motion remains above this desired trajectory (more flexed in the flexion phase of swing or more extended in the extension phase), no assistance is provided. Also, unlike a conventional controller, the control torque is not the output of the PD controller, *U*_1_; rather, the output of the PD controller scales the magnitude (although not direction) of the feedforward torque pulse. The sign of the torque pulse is guaranteed to be maintained by the presence of a signum function and the subsequent saturation block. Due to the signum function, when *G*(*øt*) is commanding a positive (i.e., flexive) torque, *U*_2_ will only be positive if the knee is flexed less than the reference. Meanwhile, if *G*(*øt*) is commanding a negative (i.e., extensive) torque, *U*_2_ will only be positive if the knee is more flexed, that is, less extended, than the reference angle. *U*_2_ is then saturated between 0 and an upper bound to produce *U*_3_, which is a gain on the torque function *g*, generating the commanded torque *U.* The result is a controller that provides users with an assist-as-needed torque pulse, which has a unique and predictable zero crossing, and a known maximum amplitude.

**Figure 3.**
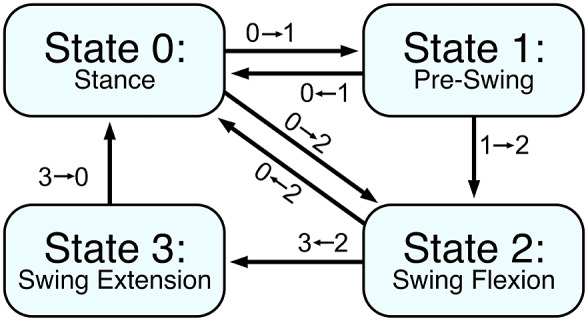
Finite-state machine used for walking. The finite-state machine consists of four states: Stance, Pre-Swing, Swing Flexion, and Swing Extension. When the device detects that the user is walking down a steep slope, the pre-swing state is bypassed to avoid knee buckling. Both swing states will transition to the Stance state upon detection of loading; a transition from Swing Flexion to Stance is possible as an exception, although not expected during typical use.

The swing-assist controller is implemented within a finite-state machine (FSM). This FSM provides transitions between the different phases of gait and generates the splines and trajectories needed for the swing-assist controller, as well as the resistance levels commanded via the hydraulic valve. The FSM employs four states, as shown in Figure 3. The conditions governing transitions between states are described in Table 1, and behaviors within each state are described below.

**Table 1.** FSM transitions

	Trigger
0 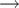 1	Thigh sufficiently behind user (indicated by angle) and not steep-slope walking
0 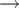 2	Steep slope walking and leg unloaded
2 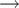 0	Leg loaded
1 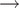 0	Thigh not sufficiently behind user prior to toe off
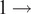 2	Leg unloaded
2 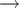 3	Knee angle trajectory velocity negative
3 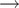 0	Leg loaded or knee reaches full extension

*Note. The conditions that identify steep-slope walking are described in the text.*

When in state 0 (stance), the hydraulic valve is set to a high damping value. This offers the user appropriate resistance against flexion during the stance phase of level walking, but also enables stance-knee yielding during steep-slope descent, as described in the following paragraph. While in this state, the drive motor bridge is fully open (i.e., zero motor current). Upon transition to state 1 (pre-swing), the valve is rotated into an open position to allow the user to begin flexing the knee. Note that this state occurs during late stance (double support), such that weight is shifted to the contralateral limb just ahead of pre-swing. During this state, passive motor control, as described in Park et al. (2016), is employed to provide controllable damping to stabilize the knee, particularly against extension (recall the hydraulic system incorporates a check valve, and therefore provides limited resistance against extension). The additional extension damping softens contact with the extension stop in cases when the knee initially loses contact with the extension stop, and is subsequently returned to it. As the leg is unloaded, the FSM transitions into state 2 (swing flexion), at which time the valve remains fully open, cadence information is calculated based on stance phase duration, and the reference knee trajectory and period scaling factor are computed. The motor torque is governed by the swing-assist controller during states 2 and 3. Once the leg has reached peak flexion, the FSM transitions into state 3 (swing extension), the valve is brought back to a locked position in advance of heel strike. Note that due to the presence of a check valve, this does not affect the extension resistance of the knee. Once the knee nears full extension, the drive motor switches back into a passive damping mode to mitigate terminal impact of the knee at the end of swing. As the leg is loaded, the FSM transitions back to state 0 (stance). The behavior of the parallel systems during each state is summarized in Table 2.

**Table 2.** FSM state behavior for normal walking

State	Hydraulic valve	Drive motor
0	High damping	Disengaged
1	Free movement	Motor damping
2	Free movement	Swing-assist controller
3	High damping	Swing-assist controller, followed by motor damping

Shallow downslope walking is typically performed in a similar manner to level walking. In the case of steep downslope walking, however, user will employ stance-knee yielding, controlled by his or her hip action. The presence of substantial stance-knee yielding – when the knee is flexed a substantial amount (e.g., >35°) while loaded, and while the thigh angle remains forward of the vertical – is used to detect steep downslope walking (i.e., downslope walking that employs stance-knee yielding). As such, the presence of a slope is not detected directly; rather, the controller detects the user’s yielding of the knee, which indicates to the controller that the user is performing a stance-knee yielding gait. When this occurs, state 1 is bypassed, since flexion for steep downslope is achieved during stance-knee yielding, and thus further flexion is not needed. Instead, the controller simply transitions from state 0 to state 2 when the leg is unloaded. Although state behaviors could remain unchanged, the controller behaviors are altered slightly to better accommodate this type of gait. Specifically, when steep downslope walking is detected, the state 0 hydraulic behavior is modified slightly to reduce stance knee resistance, and to change the resistance as a function of knee angle to compensate for the nonlinear transmission ratio of the slider crank. This provides a more constant stance-knee resistance to the user. This change is not essential for steep downslope walking, but users preferred a more uniform stance-knee resistance to one that varied. Additionally, a different extension trajectory and torque pulse are implemented, to better accommodate the different swing-phase patterns employed in steep-slope descent. It should be noted that steep downslope walking can still be performed without these behavioral changes, and therefore detection of this activity is not essential to safe functionality. Furthermore, it should be noted that steep downslope behaviors would not be problematic if employed instead of level walking behaviors. Regardless, users preferred the downslope-specific behaviors to the universal stance-phase behaviors, and the associated detection performed robustly in the experiments conducted herein.

## 4. Experimental assessment

**Figure 4.**
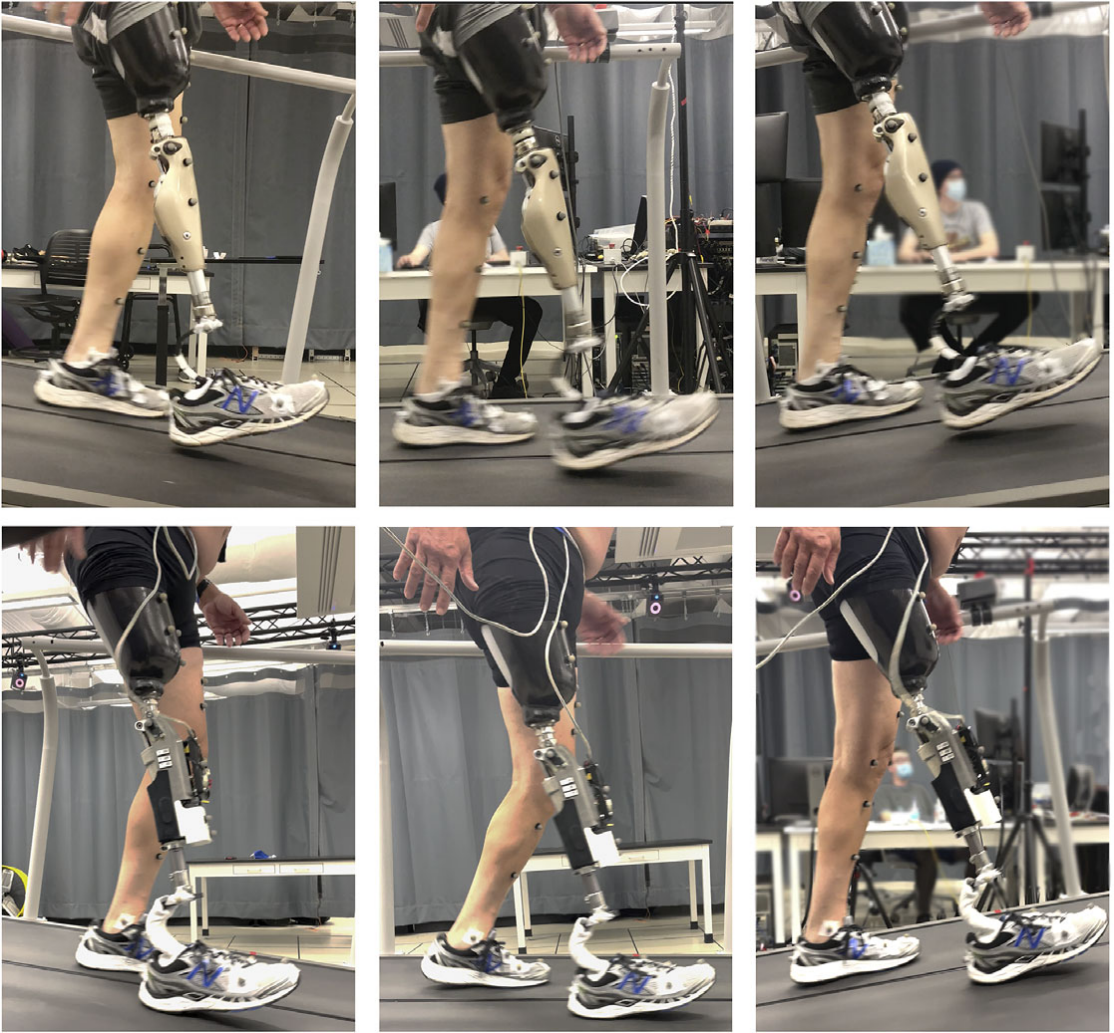
Experimental setup. All trials were performed on a treadmill while wearing lower body motion capture markers. The studies were done first on the participants’ prescribed devices, then repeated on the SCSA. Top row: Participant 1 walking at down 8°, level, and up 8° with prescribed prosthesis (C-Leg 4). Bottom row: Participant 1 performing trials with SCSA prosthesis prototype.

The previously described controller was implemented in the prosthesis prototype and tested on three participants with transfemoral amputation, with the intention of assessing the prospective benefits of the stance-controlled swing-assist approach on level, upslope, and downslope walking. For each participant, two sets of trials were performed – one with each respective prescribed prosthesis (in this case, all participants wore MPKs), and one with the swing-assist prosthesis prototype, as depicted in Figure 4. For trials with the swing-assist prototype, each participant used his daily-use socket, and each used his prescribed, passive foot prosthesis, in an effort to isolate differences resulting from the different knee units. Trials were performed on a force-instrumented treadmill (Bertec, Columbus, OH, USA) at four level walking speeds, and additionally at two upslope and two downslope grades. Use of the instrumented treadmill, along with a motion capture system (Vicon, Oxford, United Kingdom), provided both kinematic and kinetic data corresponding to the walking trials. The detailed experimental protocol is described below.

The participants’ characteristics are summarized in Table 3. Prior to the day of testing, each participant was allowed at least 1 h of walking practice on the Vanderbilt SCSA knee. When performing the experiment, the participant, first wearing their prescribed daily-use device, donned 40 motion capture markers on their lower body. The participant then walked on the force-instrumented treadmill, set to a level slope, and the treadmill speed was incrementally increased and then incrementally decreased to find the subject’s preferred (self-selected) walking speed. The participant then walked for 120 s at this speed, where the final 90 s was recorded for data processing. Due to physical limitations at higher slopes and speeds, participant 3 only performed 90 s of walking per trial, with the last 60 s worth of data being recorded for processing. After completing the level ground walking trial at self-selected speed, the trial would then be repeated at 125% of the participant’s self-selected speed, 75% of self-selected speed, and at a treadmill speed of 0.5 m/s. For participant 3, only three speeds were tested at level slope, as 75% of his self-selected speed was nominally 0.5 m/s. After completing the level walking trials, a preferred speed was identified at grades of ±4°, then again at ±8°. For each grade, the participant walked for the same duration at selected speed, where the first 30 s were not analyzed to allow for acclimation.

**Table 3.** Participant characteristics

	Age	Sex	Etiology	Years of use	Prescribed device
Participant 1	63	Male	Trauma	50 years	C-Leg 4
Participant 2	33	Male	Trauma	5 years	Genium X3
Participant 3	67	Male	Trauma	25 years	Orion Knee

After completing all trials with prescribed prostheses, each participant was allowed a break, after which their prescribed knee prosthesis was removed and replaced with the Vanderbilt SCSA knee. For all participants, their prescribed foot prosthesis was transferred between knee devices without altering plantar/dorsiflexion alignment. Once wearing the SCSA knee, the participants repeated the four-level ground trials, as well as the four sloped trials, each at the speeds selected on their prescribed knees. For all trials on the SCSA knee, the controller as described in the previous section was run on an external laptop using MATLAB Simulink Realtime. Signals from the knee were passed to the computer; and commands to minimum reference angle, feedforward torque, and motor damping were passed to the knee via CAN communications. Between participants, the amplitude and duration of the feedforward torque pulse were tuned to the individual’s preference, and the stance knee damping value was also tuned according to their weight. For all participants, this was done during the training session prior to data collection.

## 5. Data analysis

During all walking trials (each knee prosthesis at all speeds and slopes), ground reaction force and motion capture data were recorded for both the affected and unaffected leg. Subsequently, inverse dynamics were performed using Visual3D (C-Motion, Germantown, MD, USA) to generate kinetic and kinematic information for each trial. From this, direct values of knee trajectory and hip torque were taken. In addition, minimum foot clearance was calculated as described by Begg et al. (2006). When computing knee angle symmetry, each affected side peak knee angle was calculated as a percent of the average of the sound side peak knee angle for both the preceding and subsequent stride. Hip power was analyzed from stance-phase knee release (also known as knee break, indicated by the knee velocity sign change which shows the start of knee flexion) to heel strike, as this comprises the range where the hip is actively contributing to swing knee trajectory.

Statistical analysis of all data was performed on an individual basis, where the relevant metric associated with each stride constituted a single data point (e.g., knee symmetry, toe clearance, and mean hip power). Each participant had different stride length and chosen self-selected speeds, and thus, the number of datapoints varied between trials, with a mean stride count of 59 strides and a standard deviation of 19 strides. The distribution of data in each trial was tested for normality using a Kolmogorov–Smirnov test. Since much of the data were determined to be non-normally distributed, significance of differences between paired values (i.e., with the SCSA versus with prescribed device) was assessed using a Wilcoxon rank test, specifically assessing the significance of differences in medians within a 1% confidence level.

## 6. Results and discussion

**Figure 5.**
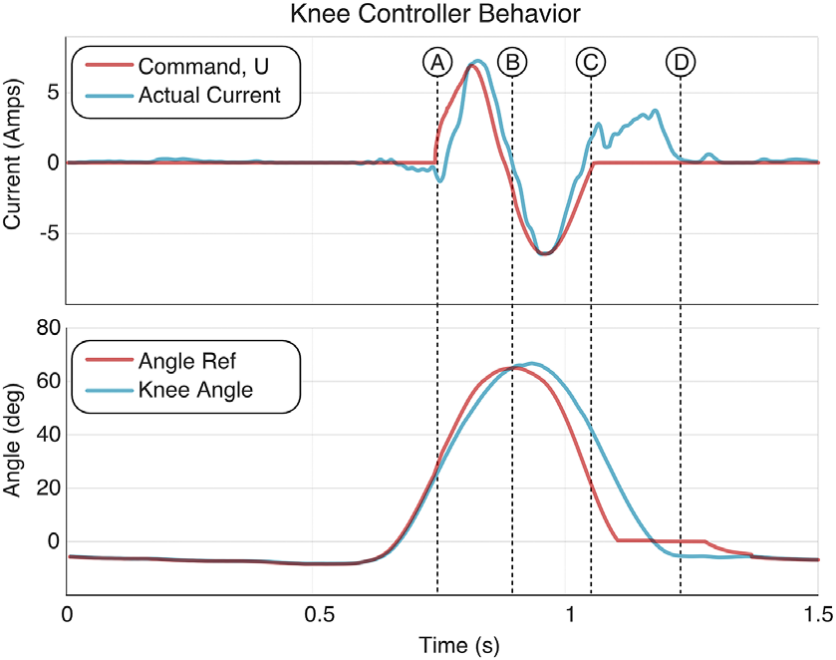
Example plot of swing-assist controller during level walking. A–D correspond to: (A) toe off; (B) transition from state 2 to state 3; (C) transition from tracking swing-assist control output to implementing motor damping (hence the flexive current at end of swing); and (D) heel strike. Note that reference angle does not affect control beyond C. This data is from a level walking trial for Participant 1.

Figure 5 shows representative controller output data from a level ground, self-selected walking trial, demonstrating the nature and level of assistance provided by the swing-assist controller. For reference, 5 A of motor assistance (as shown in Figure 5) corresponds approximately to 5 Nm of torque at the knee.

**Figure 6.**
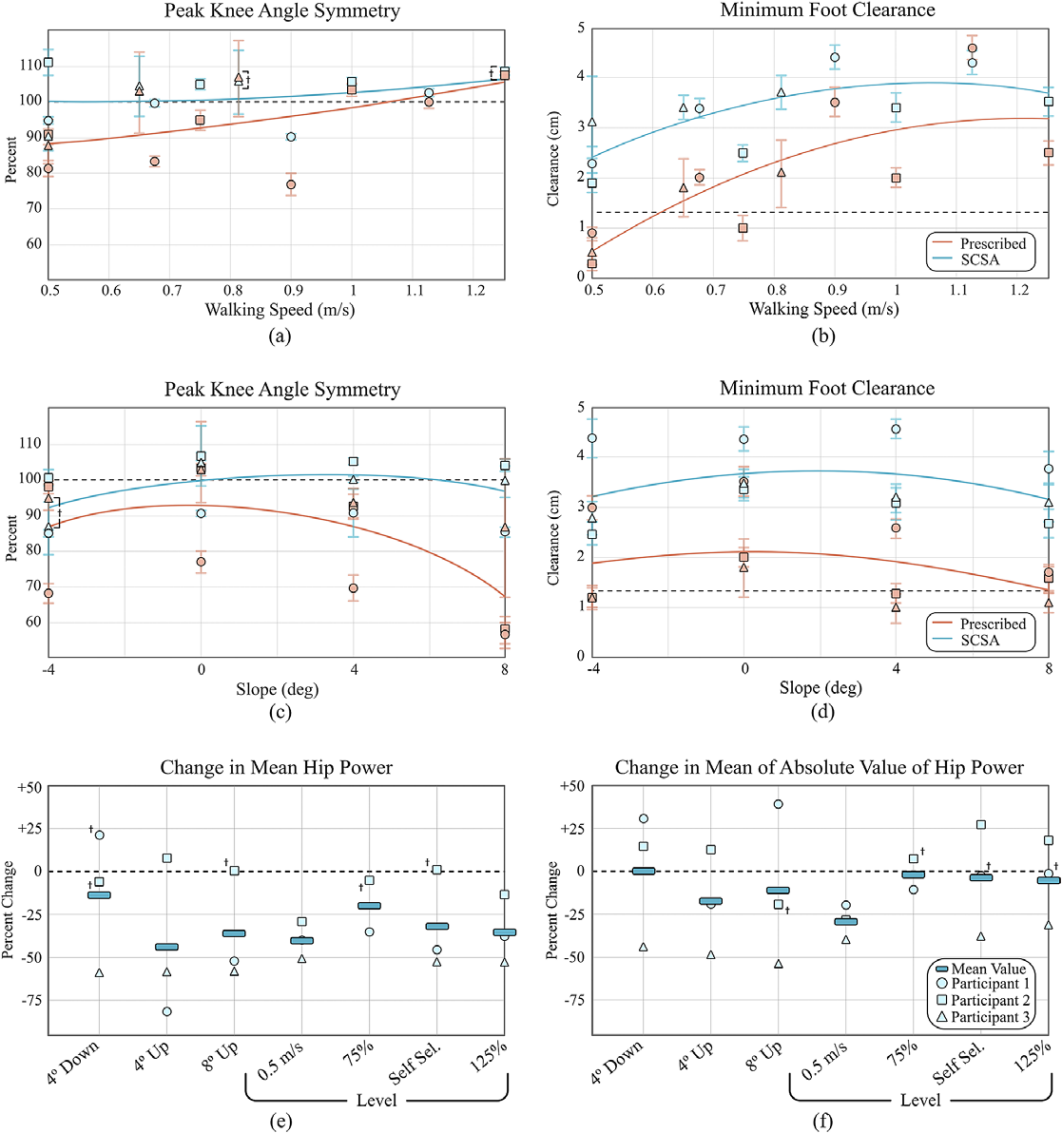
Results from experimental trials comparing SCSA prosthesis with respective prescribed prostheses: (a) Peak knee angle symmetry across different walking speeds; (b) minimum foot clearance across different walking speeds; (c) peak knee angle symmetry across different treadmill slopes; (d) minimum foot clearance across different walking slopes; (e) change in hip effort for all experiments, as measured by mean hip power from knee break to heel strike; (f) change in hip effort for all experiments, as measured by mean of absolute value of hip power from knee break to heel strike. In plots (e) and (f), the means of the three participants are indicated by bars; these bars are intended to indicate aggregate trends for purposes of visualization but are not intended to convey statistical significance. Obelisks in all plots indicate lack of statistical significance in differences between the SCSA data and the prescribed data for the points indicated. The dashed horizontal line in panels (b) and (d) indicate average toe clearances in Rosenblatt et al. (2017) which were strongly associated with occurrence of falls.

The kinematic/kinetic results of all trials are shown in Figure 6a–f, which specifically show median values and interquartile ranges (IQRs) for knee angle symmetry, toe clearance, and hip power data for the four-level ground trials, as well as down 4° and both incline trials. Down 8° data is not included in these plots, since peak flexion in down 8° walking occurs during stance phase (i.e., swing phase entails extension only). The data used to generate these plots are provided in tabular form in Supplementary Material. A video showing all trials for Participant 3 is also included as Supplementary Material.

In steep down slope descent, all three participants reported their preferred method of ambulating down 8° slopes as that described in the control section, where the knee flexes in front of the user’s body. Despite that, the prescribed devices of participants 2 and 3 displayed bimodal behavior, with participant 2’s Genium X3 forcing him to vault over the knee in 9% of strides, and participant 3’s Orion Knee forcing him to vault over in 61% of strides. For all participants, the SCSA knee behaved unimodally, allowing the correct behavior in all strides.

### 6.1. Variable speed walking

Figure 6a,b show the median and IQR of peak knee angle symmetry and minimum foot clearance for each participant across walking speeds for both the SCSA device (shown in blue) and prescribed (shown in red) devices. The plots also include second-order least squares trend lines through the SCSA and prescribed data, respectively. As indicated by the Figure 6a, the swing-assist device resulted in larger affected peak knee angles relative to sound side for all but one of the level ground trials. Note that all differences between medians were significant, unless otherwise noted by asterisks. It should be noted that some of the trials which are statistically significantly different appear similar within the plots below. This is a result of the non-normal distribution of data and graphically symmetric representation of IQR, which in some cases make these distributions appear more similar than they are. While there was variation between participants, the SCSA approach generally resulted in peak knee angles notably more symmetric between sides, as indicated by the second-order best-fit curves shown in Figure 6a. It is potentially worth noting that while in multiple cases, the SCSA control resulted in a higher peak knee angle on the affected side than on the sound limb, the controller was tuned to user preference. Increased symmetry could alternatively be enforced if desired. As mentioned earlier, however, most prosthetic feet do not provide dorsiflexion during swing, and therefore some increase in prosthetic side knee flexion relative to sound side knee flexion may be desirable.

As a result of generally increased knee flexion, the participants also achieved significantly increased minimum foot clearances when using the SCSA, as is shown in Figure 6b. On a trial-by-trial basis, minimum foot clearance was increased for all participants in all level trials, with the exception of Participant 1’s fast walking trial (1.125 m/s). As a result, the average stride was presumably less likely to incur a scuff or stumble across speeds, as can be seen from the second-order polynomial fit lines in Figure 6b. Most notably, however, at the lowest speed, minimum median clearance was increased from 0.9, 0.3, and 0.5 cm, to 2.3, 1.9, and 3.1 cm in Participants 1, 2, and 3, respectively. During data acquisition at this speed, both Participants 2 and 3 scuffed their feet multiple times on the belt during the trial when using their prescribed devices, while no scuffs were observed during testing with the SCSA.

### 6.2. Variable slope walking

Figure 6c,d shows the peak knee angle symmetry and minimum foot clearance across treadmill slopes for both the SCSA device and prescribed devices. As shown in Figure 6c, in general, the participants achieved more symmetric knee angle peaks with the SCSA than with their prescribed devices. Note that all differences between medians in Figure 6c were significant, except where indicated with an asterisk. Symmetry was especially improved on inclines, where ballistic movements become progressively less capable of producing enough motion at the knee. While not tested in this work, this trend would likely continue as inclines continued to increase, with MPK users gradually progressing from a bilaterally-symmetric gait to a step-to-gait at steep inclines. As indicated in Figure 6d, the SCSA knee also allowed users to achieve significantly higher minimum foot clearances across grades, relative to respective prescribed prostheses.

### 6.3. Hip effort analysis

To evaluate if the increase in knee movement and toe clearance was due to increased hip effort, hip power was analyzed for all trials. Hip data for each stride was analyzed from stance-phase knee release (when the knee begins to flex, but the foot is still on the ground) through heel strike, since this is the phase of gait where the SCSA’s behavior differs from that of a standard MPK. To bracket the hip effort, two measures of hip power were used: 1) the percent change in mean hip power per stride, and 2) the percent change in the mean of the absolute value of hip power per stride. The former assumes negative power offsets positive power while the latter assumes both negative and positive power contribute equally to hip effort. It is likely that some dissipative energy will be stored in tendons and subsequently returned, and as such a more accurate measure of exertion will likely lie somewhere between these two proxies for hip effort, though it is beyond the scope of this work to estimate where.

Figure 6e,f show plots of the relative mean hip power and the relative mean of absolute value of hip power, respectively, across all trials, where each point represents the median change across strides for each participant. Percent changes in medians that were not statistically significant are indicated in the plots by an asterisk. As such, an asterisk in Figure 6e,f indicates that the hip power was not significantly different between the SCSA case and the prescribed device for the respective condition and participant. Since least squares trend lines are not suited to these plots (i.e., the conditions mix speeds and slopes), the mean of the three medians for each condition was added to each plot as horizontal bars to represent the aggregate trend in the data. Averaged across participants, trials resulted in a slight reduction of requirements of both mean hip power and mean of absolute value of hip power, as indicated by the bars in Figure 6e,f, respectively. On an individual basis, participant 2 tended to have increased absolute value of power but no notable change in average power, whereas participants 1 and 3 generally showed a decrease in both metrics. One potential source of this inter-subject variability is alignment of daily use devices. For all three participants, the SCSA was aligned to be slightly hyper-extended, while participants 1 and 3 had their daily use device nominally neutrally aligned, and participant 2 had his device aligned flexed (roughly 14° flexed during stance). These results indicate that the improvements to knee symmetry and toe clearance are likely the result of the swing assistance in the SCSA, rather than additional hip effort on behalf of the users.

### 6.4. Subjective feedback

In this study, no subjective feedback was formally solicited from participants; however, spontaneous feedback was given by each of the participants during the course of the trials. Participants 1 and 2 had been a part of previous studies involving this device, so they had prior exposure to the device, although not to the controller *per se.* As such, neither spoke to the general abilities of the prototype, but both made remarks about the intuitiveness of the new controller (i.e., the new device behavior). Participant 1 also noted that he felt that he could comfortably walk faster on the prototype device, though this was not tested. Participant 3 had not used the prototype prior to the trial and remarked at the end of the study that he felt more comfortable and confident in his walking at slow speeds and on slopes on the SCSA prototype than he did on his prescribed device.

## 7. Conclusion and future work

This article proposes a swing-assist prosthesis and associated walking controller with the intent to improve walking tasks that are either entirely ballistic or bridge the gap between ballistic and non-ballistic movements. A preliminary study involving three participants with transfemoral amputation indicated that, relative to prescribed MPKs, the swing-assist prosthesis and walking controller increased the symmetry of peak knee angle across walking speeds and slopes, and similarly increased foot clearance. It is reasonable to suggest that such an increase in foot clearance offers users a gait, which, all else being equal, is less likely to be hindered by scuff or stumble perturbation. Analysis of hip power during these trials indicated that the observed differences were likely due to the difference in knee prosthesis, rather than user effort. That is, for nominally equivalent inputs at the hip, users experienced increased minimum foot clearance when using the SCSA device. The results presented herein indicate that swing-phase assistance may offer benefits during ambulation, particularly with respect to robustness and safety, when compared to energetically passive devices.

## Data Availability

The data for this publication have not been made public as they are part of ongoing research. Data can be provided to individuals upon reasonable request.

## Supplementary material

For supplementary material accompanying this paper visit https://doi.org/10.1017/wtc.2023.4. click here to view supplementary material
