# Supplementary figures and images for: The effects of swing assistance in a microprocessor-controlled transfemoral prosthesis on walking at varying speeds and grades
Source: Wearable Technol. 2023 Mar 2;4:e9. doi: 10.1017/wtc.2023.4 (PMC10936271; doi:10.1017/wtc.2023.4)

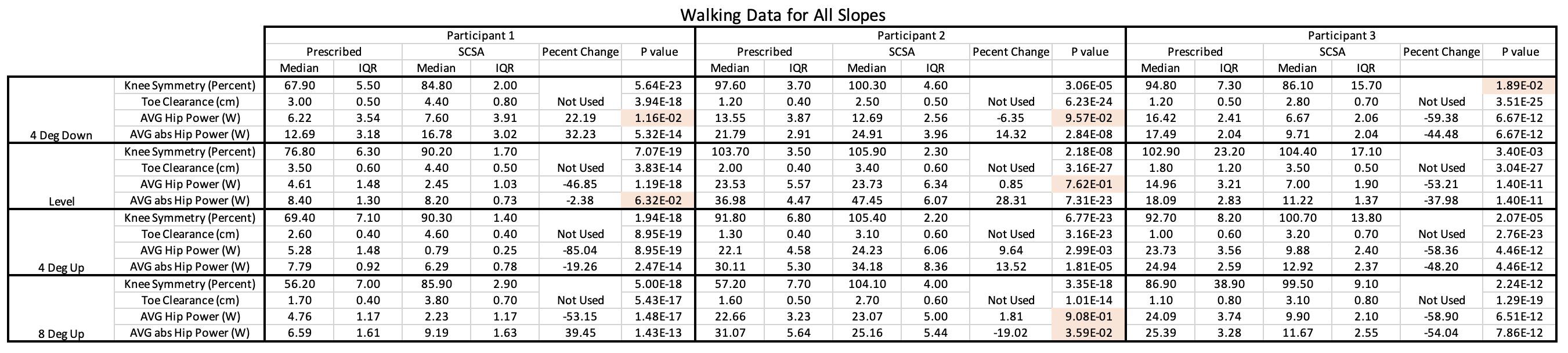

Supplement: Supplementary file 1 [file wtcsup.zip › S263171762300004Xsup001.png]

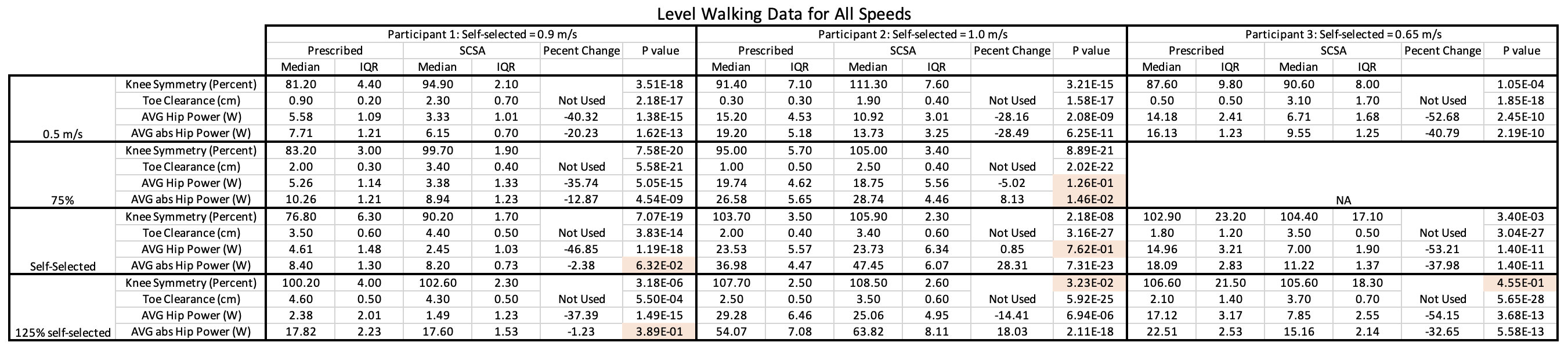

Supplement: Supplementary file 1 [file wtcsup.zip › S263171762300004Xsup002.png]
